# Cerium oxide nanoparticles protect against chondrocytes and cartilage explants from oxidative stress *via* Nrf2/HO-1 pathway in temporomandibular joint osteoarthritis

**DOI:** 10.3389/fbioe.2023.1076240

**Published:** 2023-02-03

**Authors:** Liping Xiong, Han Bao, Size Li, Deao Gu, Yuyang Li, Qianwen Yin, Wen Li, Leiying Miao, Chao Liu

**Affiliations:** ^1^ Department of Orthodontics, Nanjing Stomatological Hospital, Medical School of Nanjing University, Nanjing, China; ^2^ Department of Cariology and Endodontics, Nanjing Stomatological Hospital, Medical School of Nanjing University, Nanjing, China

**Keywords:** cerium oxide nanoparticles, temporomandibular joint, osteoarthritis, oxidative stress, Nrf2/HO-1 signal pathway, cartilage explant

## Abstract

Oxidative stress is closely linked to the etiology of temporomandibular joint osteoarthritis. (TMJ-OA) and is an important therapeutic target. Cerium oxide nanoparticles (CNPs) have been broadly studied owing to their powerful antioxidant properties and potential preventive and therapeutic effects against chronic diseases. The current study was designed to explore the protective effects of CNPs on the progression of TMJ-OA and their potential mechanisms. We detected the ability of CNPs to eliminate reactive oxygen species (ROS) in chondrocytes. Moreover, their protective effects on chondrocytes were detected in the level of gene and protein. Furthermore, TUNEL assay, histology and safranin O-fast green staining were used to detect the beneficial effects of CNPs on cartilage explants. The mechanism of CNPs, protecting condylar cartilage by reducing inflammation, was further explored by knocking down the Nuclear factor-erythroid 2-related factor (Nrf2) gene. CNPs could reduce the ROS levels in chondrocytes and cartilage explants and reverse the IL-1β-induced imbalance of cartilage matrix metabolism and apoptosis. The protective effects of CNPs on cartilage were lost after key antioxidant factors including Nrf2 and heme-oxygenase 1(HO-1) were significantly reduced. In conclusion, this study demonstrated for the first time that activating the Nrf2/HO-1 signaling pathway by CNPs might have therapeutic potential for TMJ-OA.

## Graphical Abstract

**Figure:**
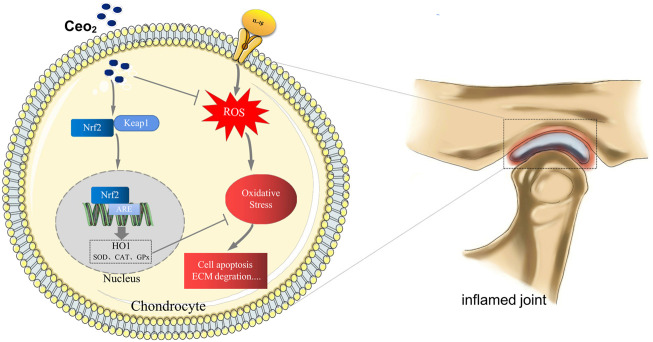
In TMJ-OA, CNPs could effectively remove ROS in chondrocytes by activating the Nrf2/HO-1 signaling pathway and exert excellent antioxidant and anti-inflammatory effects.

## 1 Introduction

Osteoarthritis (OA) is a widespread and increasingly serious joint disease, causing joint pain and disability (Martel-Pelletier et al., 2016). The temporomandibular joint (TMJ) is a hinge and gliding joint, connecting the mandibular condyle with the temporal articular surface, and is the most common joint affected by OA (Ottria L et al., 2018). TMJ-OA has a similar pathological process as that of other OA joints, including joint pain, synovitis, progressive cartilage degeneration, subchondral bone, and periarticular tissue changes. Due to the complex etiology and unique physiological anatomy of TMJ-OA, effective prevention and treatment remain challenging (Milam, 2005).

According to previous studies, oxidative stress plays an important role in the development of OA. During joint inflammation, the imbalance of intracellular redox occurs, the increased reactive oxygen species (ROS) as a signaling intermediate, further preventing ECM synthesis、cell migration and growth factor bioavailability; activating the matrix metalloproteinases, directly degrading of ECM components; increasing chondrocyte apoptosis and ultimately aggravating the progression of TMJ-OA (Poulet and Beier, 2016; Bolduc et al., 2019). Under normal physiological conditions, the enzyme-dependent and enzyme-independent endogenous antioxidants protect the organism from oxidative damage. However, the excess of free radicals can disrupt the capability of intracellular antioxidant defenses, leading to persistent tissue damage (Gorrini et al., 2013; Poljsak et al., 2013). Nuclear factor-erythroid 2-related factor (Nrf2) can regulate the cellular antioxidant responses and expression of genes related to the cellular oxidative defense system. In response to oxidative or electrophilic stress, Nrf2 can dissociate from keap1 the cytoplasm and translocate to the nucleus, where it binds to antioxidant response elements (AREs) and activates the transcription of various antioxidant genes, such as heme-oxygenase 1 (*HO-1*), which are known for their ability to counteract ROS(Alcaraz and Ferrándiz, 2020). A study on experimental arthritis indicated that Nrf2 was a major factor in controlling oxidative stress and joint degradation. Nrf2 deficiency could exacerbate the arthritic process, while the over expression of *HO-1* exerted fascinating antioxidant, anti-inflammatory, and anti-catabolic effects, and activation of the Nrf2/HO-1 signaling pathway displays potent protective effects in TMJ-OA chondrocytes and animal models (Jiang et al., 2020). Based on this observation, numerous therapeutic approaches, such as antioxidant supplements, mediators of various ROS pathways, and free radical scavengers, have been used to treat TMJ-OA. Although small molecules, including curcumin and resveratrol have better antioxidant effects, they have poor water solubility, short half-life, unsustainable efficacy, and low bioavailability (Dulbecco and Savarino, 2013; Ren et al., 2021). On the other hand, natural enzymes have inherent limitations, such as high cost, low stability, and storage issues, leading to their unsustainable use (Wu et al., 2019). In addition, the challenges in effective drug delivery and drug release profiles have limited the successful exploration of controlled release systems for some OA drugs (Kavanaugh et al., 2016).

Currently, cerium oxide nanoparticles (CNPs) are gaining popularity as inorganic nanomedicines in antioxidant therapy (Saifi et al., 2021). Cerium (Ce), a lanthanide element, is the most abundant among rare-earth elements. Cerium oxide is capable of rapid regenerative redox cycling between the two oxidation states of Ce, Ce^3+^, and Ce^4+^, due to the presence of oxygen vacancies in its lattice. This property makes it an interesting material to maintain its powerful catalytic activity *in-vivo* for a long time, requiring less repeated doses. Moreover, at the nanoscale, the increased surface area to volume ratio and altered quantum lattice of CNPs further enhance their specific redox activity. Due to their excellent anti-oxidation properties, CNPs have attracted considerable attention in biomedical applications. In previous studies, CNPs have shown great potential for the treatment of cancer, Alzheimer’s disease, liver inflammatory disorders, acute kidney injury, radiation-induced tissue damage, retinal disease, and constipation (Montini et al., 2016; Sadidi et al., 2020; Wu and Ta, 2021). However, to the best of our knowledge, studies investigating the effectiveness of CNPs for TMJ-OA are lacking.

The current study hypothesized that CNPs could inhibit the TMJ-OA-induced degeneration of articular cartilage by enhancing the antioxidant function of chondrocytes. Chondrocytes and cartilage explants were exposed to the *in-vitro* microenvironment of IL-1β-induced TMJ-OA to verify the protective effects of CNPs. Moreover, this study also explored the underlying mechanisms of these protective effects, involving Nrf2/HO-1 signaling pathways.

## 2 Materials and methods

### 2.1 Chemicals and reagents

The following chemicals and reagents were used in this study: Ce(NO_3_)_3_.6H_2_O (Sigma, United States, 202991), ethylene glycol (Aladdin, China, E103321), ammonium hydroxide solution (Sangon, China, A530001), citric acid monohydrate and trisodium citrate dehydrate (Reagent, China, 10007118, 10019418), sod assay kit (Dojindo, Japan, S311), hydroxyl radical assay kit (Nanjing Jiancheng, China, A018), the CheKine™ catalase (CAT) activity assay kit (Abbkine, China, KTB1040), phosphate buffer saline (PBS; Hyclone, United States, SH3025601), 0.25% trypsin-EDTA (Gibco, United States, 25200056), 0.2% type II collagenase (Sigma, United States, 9001-12-1), dulbecco’s modified eagle medium (DMEM; Gibco, United States, C11995500BT), fetal bovine serum (FBS; Gibco, United States, 10099141), penicillin and streptomycin (Gibco, United States, 15140122), 1% osmium tetroxide (Agar Scientific, UK, AGR1019), epoxy resin (Flight biotech, China, GP2001),1% lead citrate (Delta, China, 6159440), 2.5% glutaraldehyde fixative solution (Phygene, China, PH9003), cell counting kit-8 (CCK-8; Dojindo, Japan, CK04), DCFH-DA (Beyotime, China, S0033S), griess reagent (Abbkine, China, KTB1400), superoxide anion assay kit (Biosharp, China, BL880A), Annexin V-FITC/PI kit (Beyotime, China, C1062S), 4% paraformaldehyde (Boster, China, AR1068), 0.5% TritonX-100(Beyotime, China, P0096), 5% bovine serum albumin (Beyotime, China, P0260), Alexa Fluor^®^488 (Invitrogen, United States, A20181), DAPI(Solarbio, China, C0060), Lipofectamine 2000 (KeyGEN, China, KGD032), TRIzol reagent (Vazyme, China, R401-01), reverse transcription kit (Vazyme, China, R212-01), SYBR-green real-time PCR master mix (Vazyme, China, R223-01), RIPA lysis buffer (Thermo Fisher, United States, PI89900), enhanced chemiluminescence kit (ECL,Vazyme, China, E412-01), TUNEL reagent (Invitrogen, United States, C10617).

### 2.2 CNPs synthesis and characterization

The synthesis of CNPs has been described in detail in our previous study (Yu et al., 2022). The particle size and morphology of CNPs were evaluated using transmission electron microscopy (TEM, JEM-2100, JEOL, Japan). In order to reduce the agglomeration phenomenon during CNPs detection, the obtained CeO_2_ solution was dispersed in water and sonicated for 1 h. The solution was then added dropwise onto a copper mesh (with a 20-nm thickness of carbon support attached to the mesh) using a dropper and left to dry for observation. Moreover, a small amount of CeO_2_ solution dispersed in water was diluted. About 1/3–2/3 of the liquid was put in the cuvette, and the hydrated particle size and distribution of CNPs were measured using dynamic light scattering (DLS, Nanosizer ZS90, Malvern, UK). X-ray photoelectron spectrometer (PHI-5000 Versaprobe III, ULVAC-PHI, Japan) was used to detect the valence distribution of Ce in CNPs. After freeze-drying the sample, a certain amount of powder was taken under the XPS for detection. The binding energy of C1s (285eV) was used as the internal reference for all measurements.

### 2.3 Enzyme mimetic activities assays of CNPs

The superoxide dismutase (SOD)-mimetic activity of CNPs was assessed according to the kit instructions. Highly water-soluble tetrazolium salt WST-1 reacts with O2- to produce a water-soluble coloured product which could be detected using a microplate reader (SpectraMax M3, Molecular Devices, United States), whereas the SOD could inhibit the reduction reaction of O_2_^−^ with WST-1, the SOD-mimetic activity of CNPs was calculated by reading the absorbance at 450 nm.

The ^•^OH-scavenging activity of CNPs was detected with a hydroxyl radical assay kit. The amount of H_2_O_2_ was directly proportional to the amount of ^•^OH produced by the Fenton reaction. When an electron acceptor was provided, a red substance, directly proportional to the amount of ^•^OH, was produced by the Griess reagent. Thus, the absorbance value at 550 nm reflected the quantity of ^•^OH present.

The peroxidase activity of CNPs was determined using the CheKine™ catalase (CAT) activity assay kit. In the presence of a suitable H_2_O_2_ concentration, CAT could react with methanol, producing formaldehyde, which reacted with the chromogenic substance. The absorbance of the product could be measured at 540 nm.

### 2.4 Isolation and culturing of primary rat condylar chondrocytes

Three-week-old male Sprague Dawley rats were obtained to extract primary chondrocytes. Cartilage tissues were removed from the TMJ, which was then dissected, rinsed with PBS, and subsequently treated with 0.25% trypsin-EDTA for 30 min at 37°C. After removing the trypsin solution, 0.2% type II collagenase solution was added for tissue digestion at 37°C for 2 h. Afterward, the digested cartilage tissue was centrifuged, and the cells were collected and seeded in a complete medium, containing heat-inactivated 10% FBS, 100 U/ml penicillin, and 100 U/ml streptomycin. Upon reaching 80%–90% confluence, the cells were passaged and transferred to culture flasks. The chondrocytes obtained from the third passage were used in this study. All the animal-related experiments were approved by the Animal Ethics Committee of Nanjing University under the ethical approval number IACUC-D2102066, and were strictly adherence to the National Institutes of Health guidelines.

### 2.5 Cellular uptake of CNPs

The ability of chondrocytes to uptake CNPs was detected using TEM. The chondrocytes were co-cultured with CNPs(160 μg/ml) for 4 h and were then gently removed from the culture dish into 1.5 ml EP tubes using a cell scraper. The sample in the EP tube was then centrifuged at 1000 rpm for 5 min, and the supernatant was carefully removed. Then, 1 ml of 2.5% glutaraldehyde fixative solution was slowly added and left for 2 h, subsequently the sample were fixed with 1% osmium tetroxide for 1 h, and gradient dehydration was performed by adding 30%, 50%, 70%, 90%, and 100% (v/v) dehydrating agents, respectively. Then, different concentrations of epoxy resin were added for infiltration, polymerized and embedded in an oven at a gradient temperature, and the cell pellet was cut into ultra-thin sections of 60–80 nm, followed by staining with uranyl acetate and 1% lead citrate, and the sections were dried at room temperature overnight and finally observed under TEM.

### 2.6 Cellular viability

The effects of CNPs on cellular viability in chondrocytes were evaluated using cell counting kit-8(CCK-8), according to the manufacturer’s instructions. The chondrocytes with a density of 5×10^3^ per well were incubated into a 96-well plate for 12 h. Different concentrations of CNPs were then added to the 96-well plate at 24 and 48 h. After washing with PBS, a 90 μl DMEM and 10 µl CCK-8 reagent were added to each well and incubated at 37°C for 2 h. Then the absorbance of each well at 450 nm was measured using a microplate reader.

### 2.7 Measurement of intracellular ROS

The inhibitory effects of cellular oxidative stress by CNPs was detected by confocal microscopy (ECLIPSE Ti2,Nikon, Japan) and flow cytometry (FACSCalibur, BD FACSCaliBur, United States) after treatment of cells with DCFH-DA probe, respectively. A cell-permeable probe was used to detect the intracellular ROS. For this purpose, a total of 1 × 10^4^ cells were incubated in a confocal petri dish for 12 h and treated with CNPs (160 μg/ml) and IL-1β (10 ng/ml). Then, the chondrocytes were incubated with DCFH-DA for 30 min and gently washed with PBS to remove unbound DCFH-DA. The levels of intracellular ROS were measured using laser confocal microscopy. Moreover, a portion of the treated chondrocytes were collected and suspended in diluted form in DCFH-DA, cells were assayed for ROS levels using flow cytometry at 488 nm after incubation and adequate washout.

### 2.8 Measurement of NO and O_2_^−^

After treatment, culture supernatants of cells and cartilage explants were collected to detect the content of NO and O_2_^−^ to further assess the ability of CNPs to eliminate ROS. The amounts of nitrite and nitrate were measured to deduce the amount of NO. Diluted standards and samples were added to EP tubes, and then working solution was added to each sample and standard tube. The reaction system was incubated at 37°C for 30 min and then 250 μl of liquid was transferred to micro glass cuvette respectively, and the OD value was read at 540 nm using UV spectrophotometer (Lambda 950, PerkinElmer, United States), and the standard curve was plotted with the concentration of standard solution as the x-axis and ΔOD_Standard_ as the y-axis. TheΔOD_Sample_ was brought into the equation to get the x-value which is the NO content.

The superoxide anion (O_2_^−^) reacted with hydroxylamine to produce NO_2_^−^, and NO_2_^−^ under the action of p-aminobenzenesulfonic acid and α-naphthylamine to produce a pink azo dye, which has the maximum light absorption at 540 nm. The samples and the corresponding reagents were added into the centrifuge tubes according to the instructions, and the reaction mix was added after 10 min at 37°C, and the OD value was measured at 540 nm in a 1 ml glass cuvette immediately after 5 min, and the NO_2_^−^ content was calculated according to the given standard curve equation.

### 2.9 Cell apoptotic assay

The cellular apoptotic assay was performed using an Annexin V-FITC/PI kit. Chondrocytes were cultured at 37°C for 24 h and washed with PBS, followed by centrifugation and resuspension in 200 μl binding buffer. Then, 10 μl annexin V-FITC and 5 μl PI solutions were added to the cell suspension and mixed thoroughly. The mixture was then kept in dark for 15 min, and 300 μl binding buffer was then added. Apoptosis was analyzed using flow cytometry at 488 nm. Total apoptosis rate is defined as the ratio of the sum of the number of early apoptotic, late apoptosis and necrotic cells to the total number of cells.

### 2.10 Immunofluorescence

The chondrocytes were seeded on a confocal small dish and pretreated with or without CNPs (160 μg/ml) for 4 h. Then, the cell cultures were co-incubated with or without IL-1β (10 ng/ml) for 24 h, followed by washing with PBS three times and were fixing with 4% paraformaldehyde. Then, the chondrocytes were subsequently treated with 0.5% TritonX-100 and blocked with 5% bovine serum albumin for 10 min, respectively. Followed by incubating with a primary antibody (Nrf2) overnight at 4°C. Then, the chondrocytes were incubated with Alexa Fluor^®^488-labeled conjugated secondary antibodies for 1 h at 37°C. The nuclei of chondrocytes were stained with DAPI for 5 min. Finally, each sample was observed under a laser confocal microscopy.

### 2.11 Small-interfering RNA (siRNA) transfection

Nrf2-siRNA was designed and synthesized by keyGEN BioTECH using the specific sequences including sense: GCAGGACAUUUGAUUTT, and antisense: AAU​CAA​AUC​CAU​GUC​CUG​CTG. Chondrocytes in log phase were taken for transfection in an enzyme-free environment according to the manufacturer’s instructions. The Nrf2-siRNA and negative control siRNA were transfected into chondrocytes using Lipofectamine 2000, cells were transfected for 6 h and replaced with antibiotic-free medium and continued to be cultured for 24 h before being used for subsequent experiments.

### 2.12 Reverse transcription-quantitative polymerase chain reaction (RT-qPCR)

Total RNA was extracted from the chondrocytes using TRIzol reagent. cDNA was synthesized using a reverse transcription kit and then amplified using a SYBR-green real-time PCR master mix. The relative mRNA expression levels of Nrf2, HO-1, Aggrecan, Collagen I, Collagen II, MMP13, ADAMST4, iNOS, COX-2, and IL-6 were calculated using the 2^−ΔΔCT^ method.

### 2.13 Western blot analysis

The total proteins were extracted from chondrocytes using pre-cooled RIPA lysis buffer, separated using SDS-PAGE, and then transferred to PVDF membranes. The membranes were then incubated with primary antibodies of Nrf2, HO-1, Aggrecan, Collagen I, Collagen II, MMP13, ADAMST4, iNOS, COX-2, and IL-6 at the specified concentrations overnight at 4°C followed by incubation with horseradish peroxidase (HRP)-conjugated respective secondary antibodies for 1 h at room temperature. After washing with TBST buffer, stripes were developed using an enhanced chemiluminescence (ECL) kit. The gray values of the stripes were quantified using ImageJ software.

### 2.14 Isolation and culturing of condylar cartilage explants

The condylar cartilage explants were isolated from the TMJ under sterile conditions and submerged into complete medium. After stabilization for 24 h, the cartilage explants were randomly grouped into wells in a 24-well culture plate and cultured with serum-free DMEM for 1 week. The cartilage explants were treated with 10 ng/ml IL-1β alone or with a combination of 10 ng/ml IL-1β and 160 μg/ml CNPs. The medium was replaced with fresh medium every 3 days. Cartilage discs were collected at the end of treatment for follow-up experiments.

### 2.15 Histopathological examination

The treated cartilage explants were fixed in 4% paraformaldehyde and subsequently paraffinized. The paraffinized explants were sectioned into 4-μm-thick sections and stained with hematoxylin-eosin (H&E) and safranin O-fast green staining solutions at the Department of Pathology, Faculty of Medicine, Nanjing University. In order to observe the apoptosis of cells in cartilage explants, the sections were stained with TUNEL reagent and DAPI, then imaged under a fluorescence microscope (ML9000F, Meizs, China).

### 2.16 Statistical analyses

All the statistical analyses were performed using one-way analysis of variance (ANOVA) with GraphPad Prism 8. All the data were obtained from at least three independent experiments and expressed as mean ± standard deviation (SD). A *p*-value <0.05 was considered statistically significant.

## 3 Results

### 3.1 Characterization and enzyme-mimicking activity of CNPs

**FIGURE 1:**
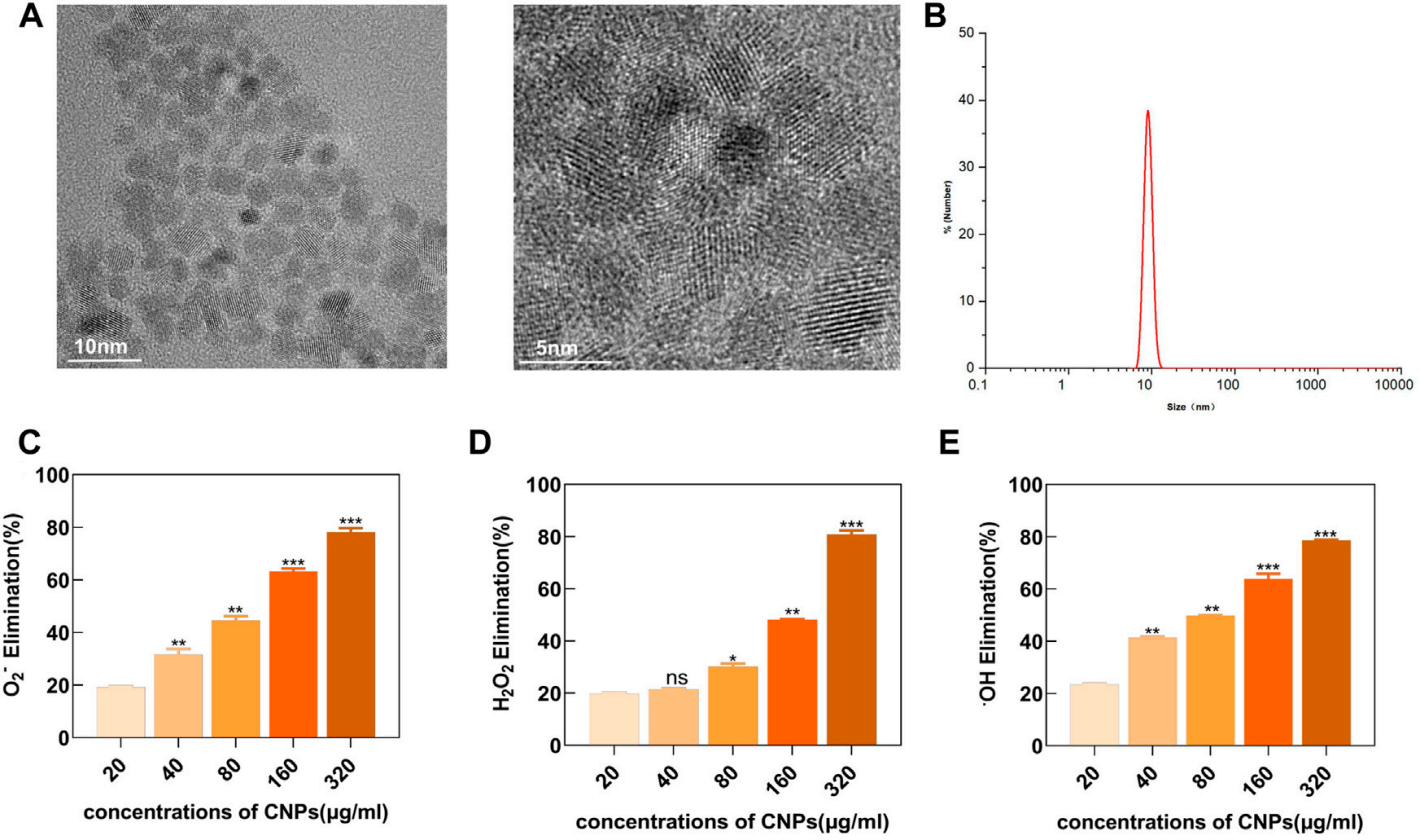
Characterization analysis and enzyme-mimicking activity of CNPs. **(A)** TEM images. **(B)** Particle size distribution of CNPs in water was analyzed using DLS. **(C)** SOD-like activity of CNPs at different concentrations. **(D)** CAT-like activity. **(E)** •OH elimination ability. ns:no significance.*p < 0.05. **p < 0.01. ***p < 0.001 relative to the 20 μg/ml group.

TEM showed that the CNPs synthesized using the low-temperature aqueous phase method had spherical morphologies with around 5 nm core sizes. Moreover, the high-resolution TEM imaging identified well-resolved lattice planes between the adjacent lattices (Figure 1A). DLS showed that the hydrate particle size was about 10 nm (Figure 1B). The XPS detection pattern shows characteristic peaks of Ce (3d) with Ce^3+^ peaks at 885.3,903.4 eV and Ce^4+^ peaks at 882.2, 898.3, 888.6 eV, and it can be found that both Ce^3+^ and Ce^4+^ are present on the surface of CNPs and the percentage of Ce^3+^ is 37.0%. (Supplementary Figure S1).

A series of *in-vitro* experiments showed that the synthesized CNPs had enzyme-like activities, mimicking SOD and CAT. As shown in (Figure 1C), an increase in the concentration of CNPs also increased the SOD-mimicking activity, indicating the effectiveness of CNPs in removing O_2_^−^. These experiments demonstrated that CNPs could also efficiently scavenge H_2_O_2_ in a dose-dependent manner (Figure 1D). In addition, H_2_O_2_ could also generate highly oxidizing •OH, which could damage the cellular viability through the Fenton reaction. The naturally occurring enzymes might not effectively scavenge •OH. However, the present study showed that CNPs could scavenge •OH in a dose-dependent manner, reaching a scavenging rate of about 80% at 320 μg/ml concentration (Figure 1E). These results indicated that the CNPs might possess powerful enzyme-mimicking activity, thereby providing a basis for the antioxidant therapy of TMJ-OA.

### 3.2 Cellular uptake and cytocompatibility of CNPs

**FIGURE 2:**
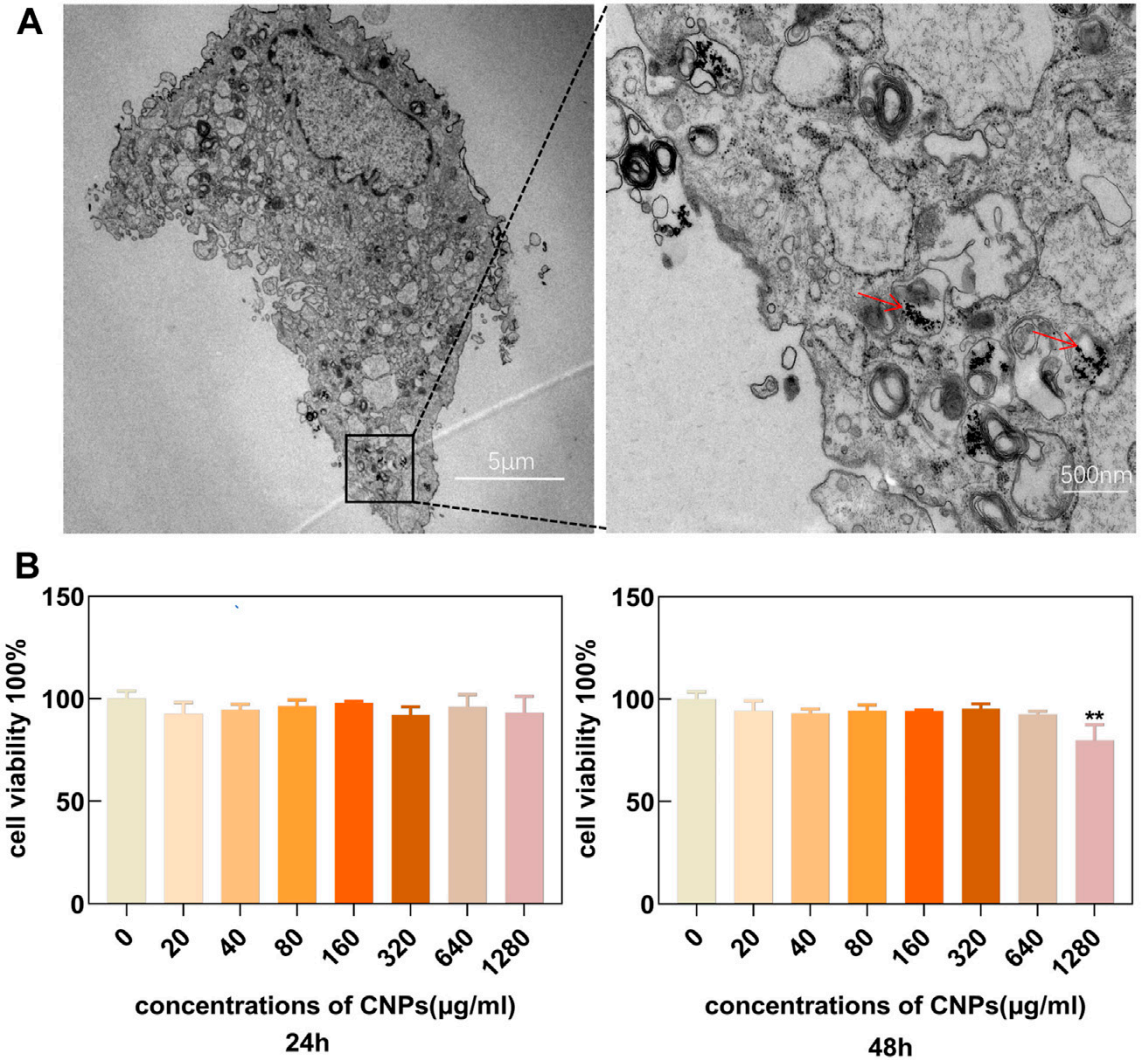
Cell uptake and cytocompatibility of CNPs. **(A)** Uptake of CNPs by chondrocytes in TEM. **(B)** Effects of different concentrations of CNPs on cellular activity after 24 and 48 h incubation with chondrocytes. CNPs are indicated by the red arrows. **p <0.01 relative to the 0 μg/ml control group.

The uptake of CNPs by chondrocytes was observed using TEM. The results showed that these cells could effectively uptake CNPs within a certain period as shown in (Figure 2A). This also ensured that the nanomaterials could enter the chondrocytes and perform their functions. The chondrocytes were cultured with different concentrations (0, 20, 40, 80, 160, 320, 640, and 1280 μg/ml) of CNPs for 24 and 48 h. CCK-8 assay showed that only the incubation of CNPs at the concentration of 1280 μg/ml for 48 h could inhibit the proliferation of chondrocytes (Figure 2B). Therefore, these results suggested that it the concentrations of CNPs below 640 μg/ml were safe for subsequent experiments. Combined with the results of *in-vitro* enzyme activity experiments (Figures 1C–E) and the effects of anti-inflammatory and antioxidant (Supplementary Figure S2), a concentration of 160 μg/ml of CNPs was selected for subsequent experiments.

### 3.3 CNPs could reduce the IL-1β-induced oxidative stress in TMJ-OA chondrocytes

**FIGURE 3:**
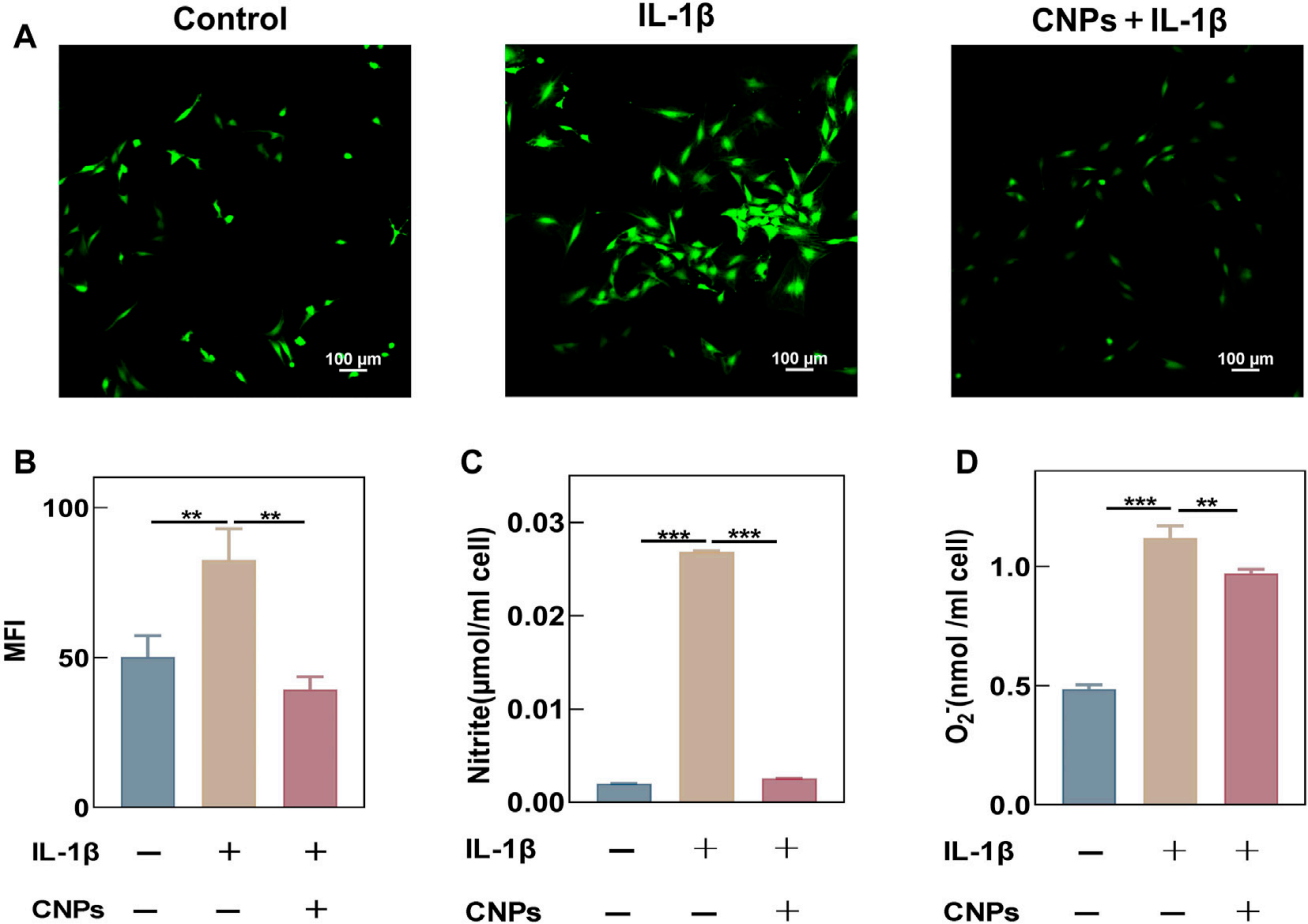
CNPs reduced the IL-1β-induced oxidative stress in TMJ-OA chondrocytes. **(A)** After pretreatment with CNPs for 6 h, the cells were stimulated with IL-1β, and a laser confocal microscope was used to detect the ROS fluorescence intensity. **(B)** MFI analysis of **(A)**. **(C)** NO contents in chondrocyte culture medium. **(D)** O_2_^−^ contents in chondrocyte culture medium. **p <0.01; ***p <0.001.

This study demonstrated that CNPs exhibited a strong antioxidant capacity. The chondrocytes were stimulated with IL-1β (10 ng/ml) to establish an inflammatory model. The ROS levels in stimulated chondrocytes were detected using a DCFH-DA fluorescent probe, and the fluorescence intensity was measured using a laser confocal microscope and flow cytometry. An increase in the intracellular levels of ROS was observed after IL-1β stimulation, while the ROS levels decreased significantly in the experimental group treated with CNPs (Figure 3A). The semi-quantitative analysis of their average fluorescence intensity also showed similar results (Figure 3B). Meanwhile, the flow cytometry experiments to further demonstrated the effect of CNPs in eliminating intracellular ROS. (Supplementary Figure S3). In addition, the contents of NO and O_2_^−^ were detected in the cell culture medium, the results once again showed that CNPs could significantly reduce the IL-1β-induced increase in the levels of ROS (Figures 3C, D).

### 3.4 CNPs could activate the expression of Nrf2 and related antioxidant genes in TMJ-OA chondrocytes

**FIGURE 4:**
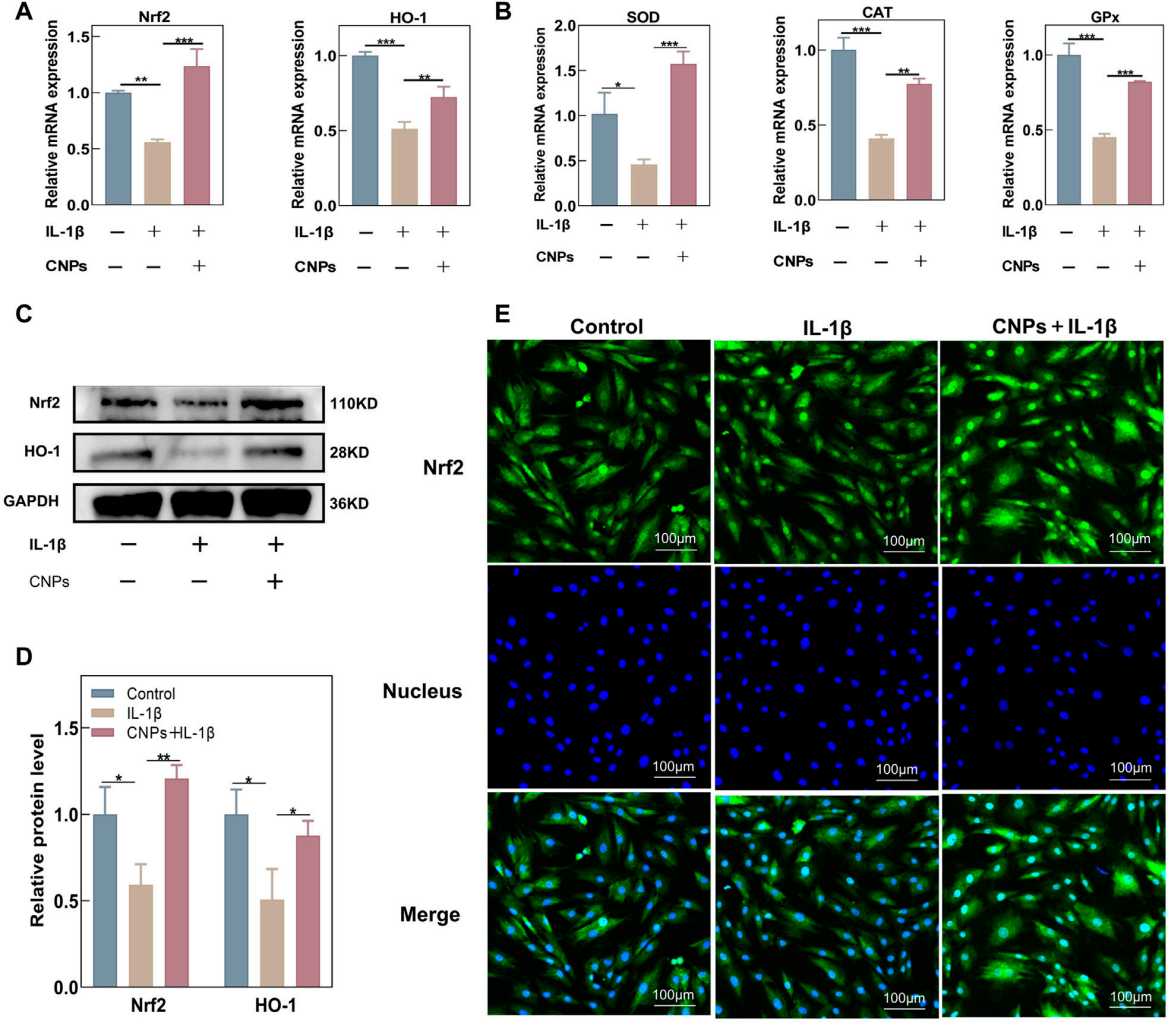
CNPs activated the expression of Nrf2 and related antioxidant genes in TMJ-OA chondrocytes. Expression of **(A)** Nrf2, HO-1, and **(B)** downstream key endogenous antioxidant genes. **(C)** Protein expression levels of Nrf2 and HO-1. **(D)** Semi-quantitative analysis of **(C)**. **(E)** Nrf2 nuclear translocation was detected using immunofluorescence (original magnification). *p <0.05. **p <0.01. ***p <0.001.

The effects of CNPs on the expression levels of endogenous antioxidant genes in chondrocytes were further explored using RT-qPCR, Western blot analysis, and immunofluorescence assays. Treating the chondrocytes with different concentrations of CNPs could significantly increase the expression levels of antioxidant key genes, including Nrf2 and *HO-1* (Supplementary Figure S4). In addition, the expression levels of Nrf2*, HO-1*, and related antioxidant genes were suppressed in the IL-1β-induced inflammatory environment; this suppression of gene expression was reversed by CNPs treatment (Figures 4A, B). Western blot analysis showed that the protein expression levels of Nrf2 and HO-1 decreased in the IL-1β-treated group as compared to the control group. However, CNPs could significantly reverse this decrease in the expression levels of Nrf2 and HO-1 (Figures 4C, D). Meanwhile, immunofluorescence staining also indicated that as compared to the IL-1β-treated and control groups, CNPs could promote the nuclear translocation of Nrf2, which is highly important for the activation of Nrf2 activity, thereby activating the expression of downstream related antioxidant genes (Figure 4E).

### 3.5 Protective effects of CNPs on IL-1β-induced apoptosis and ECM degradation in TMJ-OA chondrocytes

**FIGURE 5:**
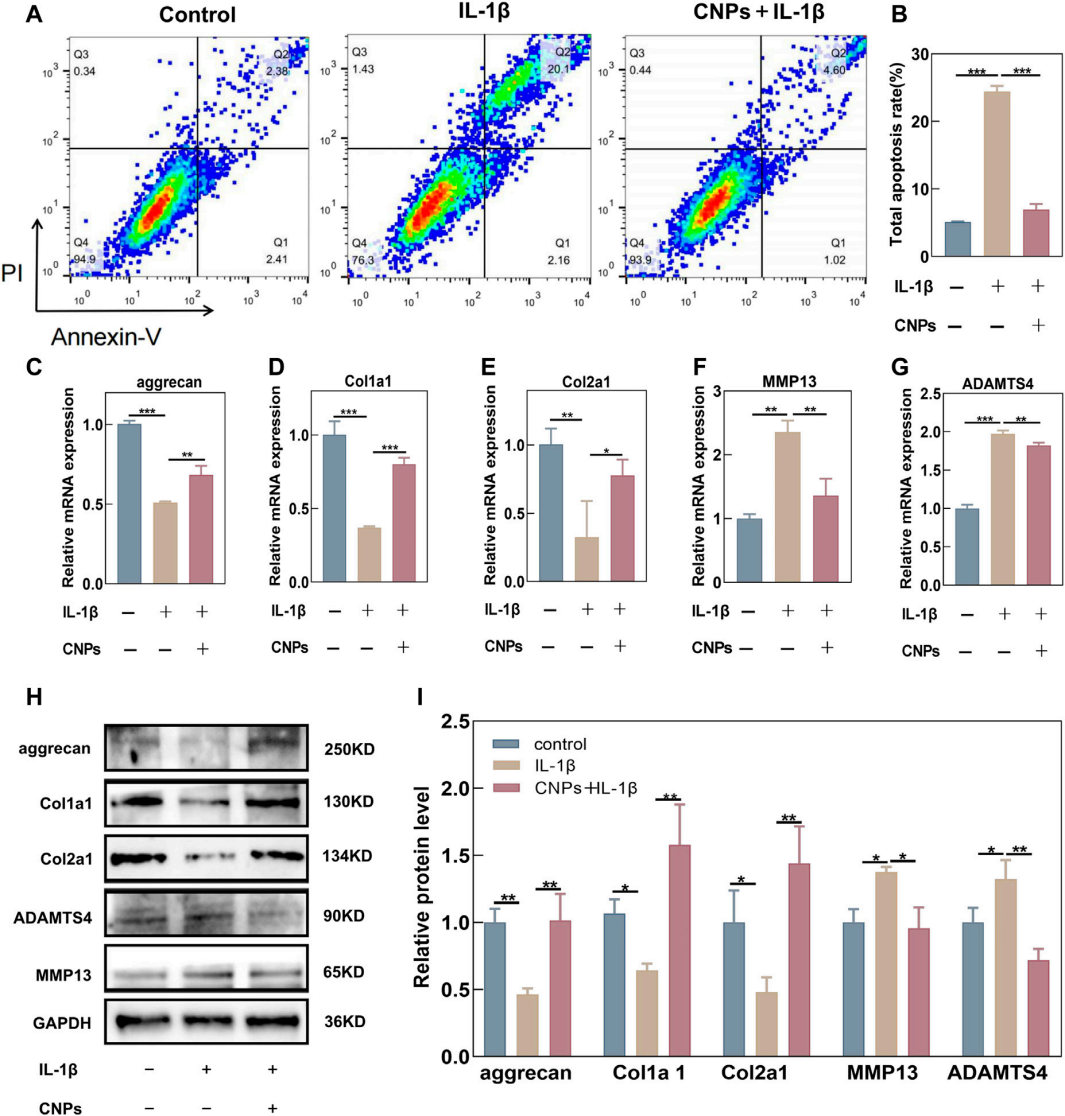
CNPs rescued the IL-1β-induced apoptosis and ECM degradation in TMJ-OA chondrocytes. **(A)** Apoptosis of IL-1β-induced chondrocytes and CNPs-pretreated chondrocytes. **(B)** Semi-quantitative analysis of the percentage of total apoptotic cells. **(C–E)** Relative mRNA expression of ECM synthesis-related genes. **(F, G)** Relative mRNA expression of ECM catabolism-related genes. **(H)** Expression of proteins related to ECM synthesis and catabolism. **(I)** Semi-quantitative analysis of **(H)**.*p <0.05. **p <0.01. ***p <0.001.

In order to investigate the protective effects of CNPs against IL-1β-induced apoptosis and ECM degradation, the number of apoptotic chondrocytes was assessed using flow cytometry, while the expression levels of ECM components were detected using RT-qPCR and Western blot analysis in the CNPs-treated and untreated chondrocytes. The results revealed an increase in the IL-1β-induced apoptosis as compared to the control group, while the CNPs treatment could reverse this increase in the IL-1β induced apoptosis (Figures 5A, B). Moreover, as shown in Figures 5C–E the synthesis of Collagen I, Collagen II, and Aggrecan, which could affect the survival of chondrocytes, was notably suppressed by IL-1β. In contrast, IL-1β could enhance the expression levels of cartilage matrix-degrading enzymes, including MMP13 and ADAMTS4, in TMJ-OA. However, pretreatment with CNPs could reverse this trend. The results of Western blot analysis were consistent with those of the RT-qPCR (Figures 5H, I). CNPs could also decrease the expression levels of inflammatory factors, including iNOS, COX2, and IL-6, in the OA chondrocytes (Supplementary Figure S5). These findings validated the protective effects of CNPs on chondrocytes in TMJ-OA.

### 3.6 Nrf2 activation was required for the protective effects of CNPs on TMJ-OA chondrocytes

**FIGURE 6:**
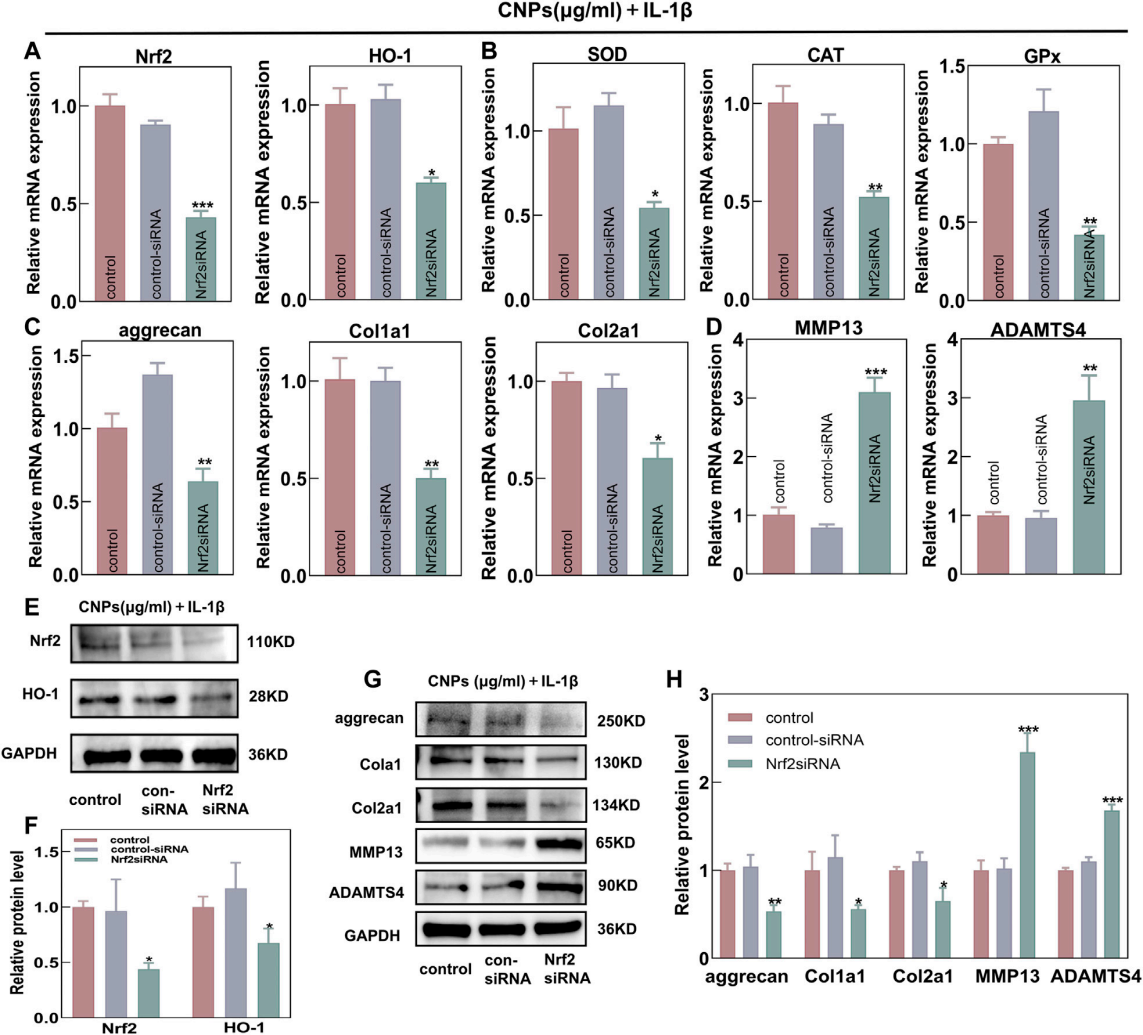
Nrf2 knockdown could eliminate the protective effects of CNPs on TMJ-OA chondrocytes. Expression of **(A)** Nrf2, HO-1, and **(B)** downstream key endogenous antioxidant genes. **(C)** Relative mRNA expression of ECM synthesis-related genes. **(D)** Relative mRNA expression of ECM catabolism-related genes. **(E)** Protein expression of Nrf2 and HO-1. **(F)** Semi-quantitative analysis of **(E)**. **(G)** Expression of proteins related to ECM synthesis and catabolism. **(H)** Semi-quantitative analysis of **(G)**. *p <0.05. **p <0.01. ***p <0.001.

In order to further explore the protective effects of CNPs on OA chondrocytes by activating the Nrf2/HO-1 signaling pathway, an Nrf2-knockdown model was established by transfecting the Nrf2-siRNA into chondrocytes and co-treating them with IL-1β and CNPs. The RT-qPCR analysis revealed that the knockdown of Nrf2 could reduce the mRNA expression levels of *HO-1* and other key antioxidant genes (Figures 6A, B). Moreover, the protective effects of CNPs on ECM under a high ROS environment were also significantly inhibited (Figures 6C, D). Western blot analysis showed similar results (Figures 6E–H). These results suggested that activating the Nrf2/HO-1 signaling pathway by CNPs could reduce oxidative stress, promote cartilage regeneration, and maintain cartilage homeostasis in the TMJ-OA chondrocytes.

### 3.7 Antioxidant and chondroprotective effects of CNPs on IL-1β-induced condyle cartilage explants

**FIGURE 7:**
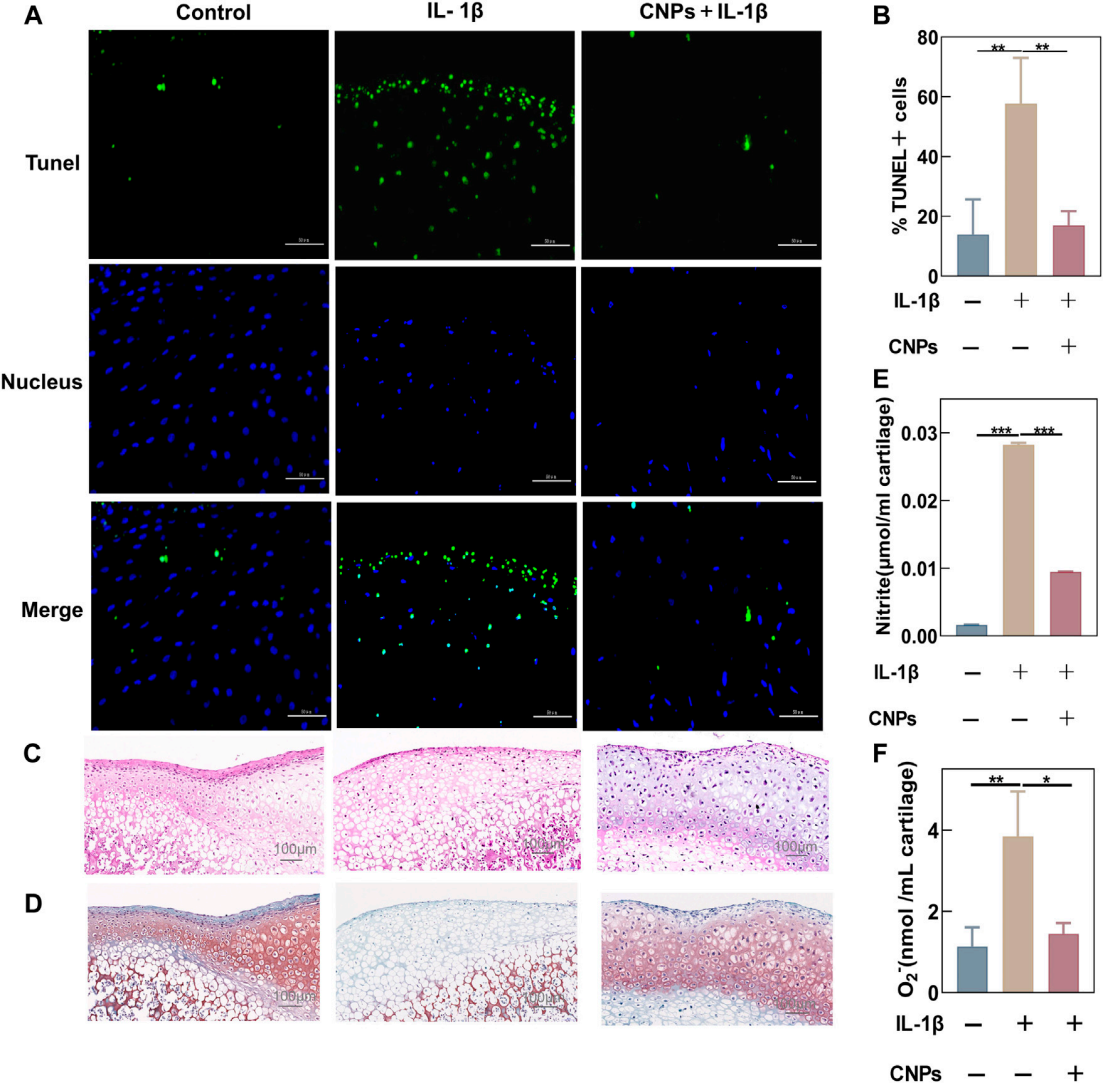
Antioxidant and chondroprotective effects of CNPs on IL-1β-stimulated condylar cartilage explants. **(A)** Apoptosis was shown by TUNEL assay after the pretreatment of cartilage explants with IL-1β stimulation and CNPs for 1 week. **(B)** Semi-quantitative analysis of the percentage of apoptotic cells. **(C)** Representative H&E staining from three groups. **(D)** Safranin O-Fast Green staining of cartilage. **(E)** Contents of NO. **(F)** Contents of O_2_^−^ in cartilage explant cultures. *p <0.05. **p <0.01. ***p <0.001.

The antioxidant capacity and protective effects of CNPs in TMJ-OA were identified by performing immunofluorescence staining of treated rat condylar cartilage explants and collecting the supernatant of the culture medium. TUNEL assay showed that CNPs could inhibit IL-1β-induced apoptosis in cartilage tissues (Figure 7A). The semi-quantitative analysis also showed that the proportion of apoptotic cells in the treatment group was lower (Figure 7B). The H&E-stained tissue sections showed that the cartilage structure was disorganized with a reduced number of chondrocytes and wrinkled nucleus in the IL-1β-stimulated group. On the other hand, comparing the number of chondrocytes in the CNPs-treated group to that in the control group showed viable ovoid chondrocytes with nuclear traps in the cartilage tissues (Figure 7C). Safranin O-fast green staining showed that the loss of proteoglycans in the IL-1β-induced group was significantly higher as compared to the control and CNPs-treated groups (Figure 7D). The NO and O_2_^−^ contents in the culture fluid of cartilage explants after IL-1β stimulation increased significantly, which was reversed by CNPs (Figures 7E, F).

## 4 Discussion

TMJ-OA is one of the most problematic joint disorders. Condylar cartilage has limited healing ability, owing to the lack of vascular system and nerves; there is no consensus treatment for its complete remission (Liu and Steinkeler, 2013; Okeson, 2013). The efficient intracellular ROS-scavenging ability of CNPs makes them a potential candidate for the treatment of TMJ-OA. Different synthetic methods have determined the physicochemical properties of CNPs. Studies have also shown that the CNPs with smaller particle sizes are catalytically more active and readily absorbed by cells (Singh et al., 2021; Baldim et al., 2018). The present study used CNPs of about 5 nm in size and spherical, which could not only effectively scavenge oxygen radicals but also avoided mechanical damage to the cells due to their round and blunt spherical structure. These efficient catalytic activities and safe biocompatibility found the bases for their applications at the cellular level.

In order to explore the antioxidant effects of CNPs, an IL-1β-induced inflammatory model was constructed. IL-1β is a potent pro-inflammatory cytokine, which is associated with tissue damage and plays a key role in developing OA by activating the production and secretion of inflammatory as well as OA pathogenesis-associated catabolic factors (Kerkhof et al., 2011). IL-1β-induced chondrocytes had horrible oxidative stress state and inflammation accompanied by a substantial increase in the intracellular ROS levels and a decrease in the expression levels of key antioxidant enzymes, including SOD, CAT, and GPx, while seriously causing the imbalance of intracellular redox status. In addition, the chondrocyte supernatant contained abnormally high NO contents, which is a highly reactive free radical and a classical pro-inflammatory factor. NO is a major inducer of chondrocyte apoptosis, and its higher concentrations have detrimental effects on chondrocyte function, thereby further exacerbating inflammatory response in the joint (Abramson et al., 2001). Due to the strong correlations between oxidative stress and inflammation, the treatment, targeting oxidative stress, might alleviate inflammation (Mittal et al., 2014; Reuter et al., 2010). A previous study reported that CNPs could alleviate the secretion of pro-inflammatory cytokines and inhibit the recruitment of macrophages in edema and allergy (Kim et al., 2021). Serebrovska et al. (2017) observed that CNPs could inhibit the lipopolysaccharide (LPS)-induced increase in the expression levels of pro-inflammatory cytokines in a rat pneumonia model, thereby showing potential anti-inflammatory and antioxidant effects. Similarly, the current study showed that CNPs could reduce the expression levels of pro-inflammatory factors by scavenging the free radicals and restoring cellular redox homeostasis, thereby alleviating oxidative stress and inflammation-induced damage to the cartilage tissues. In addition to inflammation, disturbance in the metabolism of the ECM might also contribute to cartilage destruction. An imbalance between catabolism and anabolism can lead to the loss of the cartilage matrix, which is a hallmark of cartilage deficiency, damage, and degeneration (Zheng et al., 2021; Speziali et al., 2015). The localized high ROS levels in chondrocytes are involved in cartilage matrix-degradation by inhibiting matrix formation, and/or inducing ECM degradation and expression of matrix-degrading enzymes (MMPs and ADAMTS) (Sun et al., 2021; Pan et al., 2019). In this study, CNPs could prevent joint destruction by eliminating the elevated levels of ROS, which placed chondrocytes in a gentle environment and promoted the synthesis of proteoglycan and collagen while reducing the expression levels of matrix-degrading enzymes. Moreover, the condylar cartilage of TMJ is fibrous, containing Collagen I and Collagen II, unlike the hyaline cartilage of the knee joint, which contains only Collagen II. This difference in composition allows the covering of condylar cartilage by a large number of dense collagen fibers with a higher surface roughness (Stocum and Roberts, 2018; Armiento et al., 2019). Therefore, the type of cartilage synthesis should be focused on to control OA. Surprisingly, the present study indicated that CNPs could increase the expression levels of Collagen I and Collagen II at both the mRNA and protein levels, thereby demonstrating the possibility of regenerating condylar fibrocartilage. This might also compensate for the lack of ECM cartilage protection by increasing only the expression of Collagen II.

These experiments demonstrated that CNPs could be a promising antioxidant for the treatment of TMJ-OA; however, the underlying mechanisms required further exploration. Previously, it was suggested that the catalytic mechanism of CNPs was mainly attributed to the highly reversible redox reaction of Ce^3+^/Ce^4+^, which could alter their free radicals-scavenging potential, thereby exhibiting a high similarity to natural enzymatic catalytic processes (Montini et al., 2016) However, in living systems, the signaling pathways sense, amplify, and integrate various external signals, which ultimately affect the decisions and fate of target cells (Schneider et al., 2012). Therefore, the interactions between CNPs and biological system components, that affect cellular behavior, should be explored. Various studies suggested that Nrf2 was a major factor regulating oxidative stress and joint degradation, and the activation of Nrf2 and the HO-1 signaling pathway was critical for maintaining the chondrocyte homeostasis and cartilage integrity (Marchev et al., 2017). Surprisingly, the current study showed that CNPs alone could activate the expression of Nrf2 and HO-1, significantly reverse the IL-1β-induced decrease in the expression levels of key antioxidant genes (Nrf2 and *HO-1*), and subsequently inhibit the expression of genes encoding downstream detoxification enzymes (SOD, CAT, and GPx) in chondrocytes. Similarly, immunofluorescence results showed that CNPs could significantly promote the translocation of Nrf2 into the nucleus, an essential step in gene-inducing and cytoprotective function. Therefore, it was speculated that the protective effects of CNPs on cartilage might be related to the activation of the Nrf2/HO-1 pathway. Based on these observations, the Nrf2-knockdown model was established in chondrocytes. The result showed that the Nrf2-knockdown could significantly reduce the Nrf2 and HO-1 expression levels, and relentlessly eliminate the protective effect of CNPs on TMJ OA chondrocytes in the inflammatory environment. This provided strong evidence for the involvement of the Nrf2/HO-1 signaling pathway. Although the present study verified that activating Nrf2 was the main reason for the protective effects of CNPs in TMJ-OA, the role of CNPs in eliminating ROS through catalytic reactions and synergistically resisting oxidative stress in chondrocytes by activating endogenous antioxidant systems could also not be denied. Anyway, this was the first study to demonstrate that CNPs could protect chondrocytes and cartilage explants from the inflammation-induced oxidative stress in TMJ-OA by activating the Nrf2/HO-1 signaling pathway.

This study evaluated the chondroprotective properties of CNPs on IL-1β-stimulated cartilage explants in a three-dimensional orientation. The CNPs treatment could significantly reduce the number of free radicals in the culture medium and visual cartilage breakage-related factors, including the number of apoptotic chondrocytes, disorganization of tissue structure, and substantial loss of proteoglycans from the cartilage matrix. Mutual validation of the chondrocyte and cartilage explant parts of the experiment, and the beneficial effect of CNPs on TMJ OA was futher confirmed.

Future studies should focus on finding the duration of the antioxidant effects of CNPs and maintaining the appropriate level of Nrf2/HO-1 activity in order to prevent the imbalance between intracellular ROS and antioxidants caused by the continuous downregulation of antioxidant capacity.

## 5 Conclusion

In this study, we demonstrated that CNPs inhibit condylar cartilage degeneration by exerting powerful antioxidant functions using an inflammatory model of IL-1β-stimulated chondrocytes and cartilage explants. It was also shown for the first time that this protective effect of CNPs on condylar cartilage was mainly attributed to the activation of the Nrf2/HO-1 signaling pathway. Therefore, the application of CNPs might possess tremendous potential in the clinical management of TMJ-OA.

## Data Availability

The original contributions presented in the study are included in the article/Supplementary Material, further inquiries can be directed to the corresponding authors.
