# Supplementary material for: Cerium oxide nanoparticles protect against chondrocytes and cartilage explants from oxidative stress via Nrf2/HO-1 pathway in temporomandibular joint osteoarthritis
Source: Front Bioeng Biotechnol. 2023 Feb 3;11:1076240. doi: 10.3389/fbioe.2023.1076240 (PMC9937079; doi:10.3389/fbioe.2023.1076240)
Supplement: Supplementary file 1 [file DataSheet1.pdf]

Supplementary materials

Cerium oxide nanoparticles protect against chondrocytes and cartilage explants from oxidative stress via Nrf2/HO-1 pathway in temporomandibular joint osteoarthritis

Liping Xiong[1], Han Bao[1], Size Li[1], Deao Gu[1], Yuyang Li[2], Qianwen Yin[1], Wen Li[2], Leiying Miao[2*], Chao Liu[1*]

[1]Department of Orthodontics, Nanjing Stomatological Hospital, Medical School of Nanjing University, Nanjing, 210008, China.
[2]Department of Cariology and Endodontics, Nanjing Stomatological Hospital, Medical School of Nanjing University, Nanjing, 210008, China.

Correspondence:
LeiYing Miao,Ph.D,Professor
Email: miaoleiying80@163.com

Chao Liu,Ph.D,Professor
Email: dxliuchao@163.com

Keywords: Cerium oxide nanoparticles; Temporomandibular joint; Osteoarthritis ; Oxidative stress; Nrf2/HO-1 signal pathway; Cartilage explant

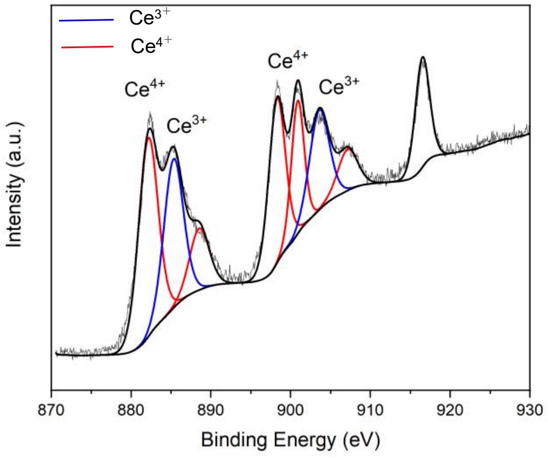

FigureS1.XPS spectrum of CNPs.

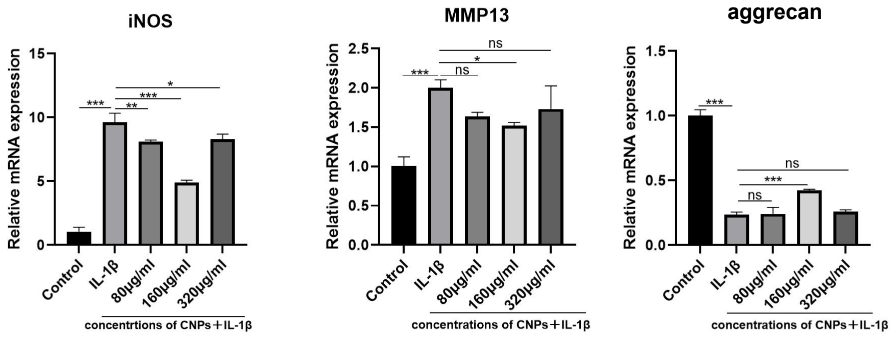

FigureS2. Effects of different concentrations of CNPs on iNOS、MMP13 and aggrecan expression levels in inflammatory states.*P<0.05. **P <0.01. ***P <0.001 relative to the control group.

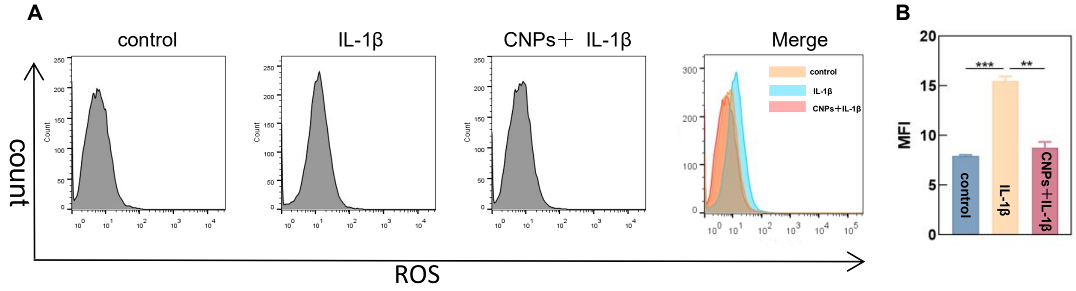

FigureS3.CNPs reduced the IL-1β-induced oxidative stress in TMJ-OA chondrocytes. (A) Flow cytometry analysis of intracellular ROS levels; (B) Semi-quantitation of intracellular ROS levels detected by flow cytometry. ***P* <0.01, ****P* <0.001.

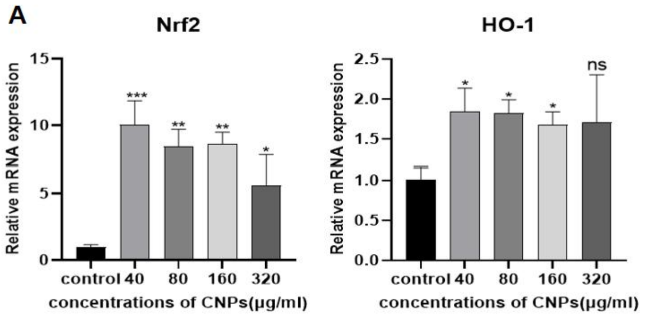

FigureS4.(A) Different concentrations of CNPs promote the mRNA expression of Nrf2 and HO-1.**P* <0.05,***P* <0.01, ****P* <0.001 relative to the control group.

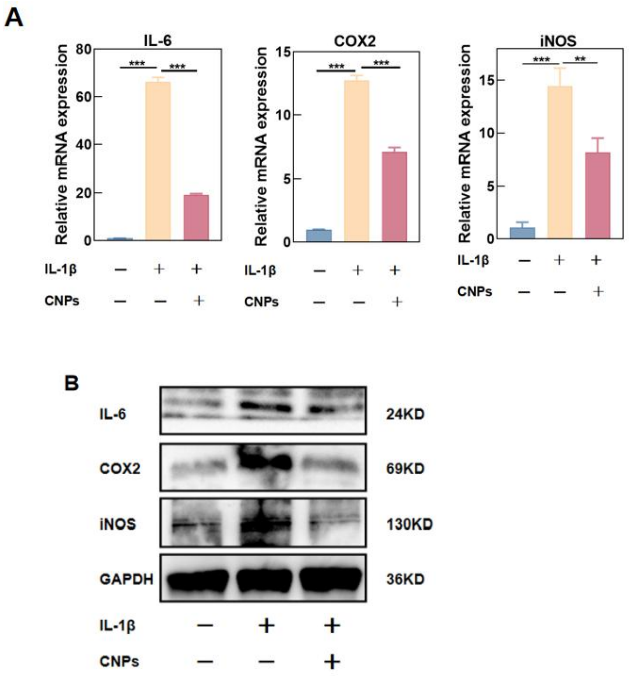

FigureS5.(A)Relative mRNA expression of IL-6,COX2,iNOS; (B) protein expression of IL-6,COX2,iNOS.***P* <0.01. ****P* <0.001.

Supplementary Table 1. Primer sequences used in this study.

Gene	Identification	Sequence (5'-3')
GAPDH	Sense	TGCCACTCAGAAGACTGTGG
	Antisense	TTCAGCTCTGGGATGACCTT
Nrf2	Sense	GGTTGCCCACATTCCCAAATC
	Antisense	CAAGTGACTGAAACGTAGCCG
HO-1	Sense	GAAGAGGAGATAGAGCGAAACA
	Antisense	CAATCTTCTTCAGGACCTGACC
SOD	Sense	CCTGTTCTACTGCAGTTAGGAA
	Antisense	CATCCTTAGCCTAGTTACACGT

CAT	Sense	AAAAGCTAACCTGTAAAGCACG
	Antisense	CTGTTAAGTGATTGCTAAGCCC
GPx	Sense	AGTGCGAGGTGAATGGTGAG
	Antisense	TCGATGTCGATGGTGCGAAA
aggrecan	Sense	CCAGAAGGGTCAGGAGAGAAC
	Antisense	AAAGTGTCCAAGGCATCCAC
Col1a1	Sense	CAAGATGGTGGCCGTTACTAC
	Antisense	TTAGTCCTTACCGCTCTTCCAG
Col2a1	Sense	GGCTCCAGAACATCACCTA
	Antisense	GCCCTCATCTCCACATCATT
MMP13	Sense	ATGTCATAACCATTCAGAGCC
	Antisense	ATCCCTTGATGCCATTACCAG
ADAMTS4	Sense	CCCGGAATGGTGGAAAGTATT
	Antisense	TATGACAAGTGCGGAGTATG
iNOS	Sense	AGCATCCACGCCAAGAACG
	Antisense	GTCTGGTTGCCTGGGAAAAT
COX2	Sense	GAGCTGTAAGAGTCTACTGACC
	Antisense	ACACAGGAATCTTCACAAATGG
IL-6	Sense	TGCACTGTCAGAAAACAATCTG
	Antisense	CCAGAGCAGATTTTCAATAGGC

Supplementary Table2. Antibodies used in this study.

MARKER (SPECIES)<br>(Primary antibodies)	DILUTION	DISTRIBUTOR/SOURCE<br>(CATALOGUE NUMBER)
GAPDH Rabbit mAb	1:5000	Abcam;ab181602
Nrf2 Rabbit mAb	1:5000	Proteintech;16396-1-AP
HO-1 Rabbit mAb	1:5000	Abcam;ab68477
aggrecan Rabbit mAb	1:1000	Affinity Biosciences； DF7561
COLI Rabbit mAb	1:1000	Proteintech;14695-1-AP
COLII Rabbit mAb	1:1000	Proteintech;28459-1-AP
MMP13 Rabbit mAb	1:1000	Proteintech;18165-1-AP
ADAMTS4 Rabbit mAb	1:1000	Affinity Biosciences;DF6986
iNOS Rabbit mAb	1:5000	Abcam;ab178945
COX2 Rabbit mAb	1:5000	Abcam;ab15191
IL-6 Rabbit mAb	1:1000	Affinity Biosciences;DF6087
